# Bioinformatics-based study reveals that AP2M1 is regulated by the circRNA-miRNA-mRNA interaction network and affects Alzheimer’s disease

**DOI:** 10.3389/fgene.2022.1049786

**Published:** 2022-11-18

**Authors:** Qi Zhang, Bishuang Chen, Ping Yang, Jipan Wu, Xinping Pang, Chaoyang Pang

**Affiliations:** ^1^ School of Computer Science, Sichuan Normal University, Chengdu, China; ^2^ West China School of Basic Medical Sciences and Forensic Medicine, Sichuan University, Chengdu, China

**Keywords:** Alzheimer’s disease, circRNA, miRNA, ceRNA, GEO, prognosis, biomarkers

## Abstract

Alzheimer’s disease (AD) is a progressive neurological disease that worsens with time. The hallmark illnesses include extracellular senile plaques caused by β-amyloid protein deposition, neurofibrillary tangles caused by tau protein hyperphosphorylation, and neuronal loss accompanying glial cell hyperplasia. Noncoding RNAs are substantially implicated in related pathophysiology, according to mounting data. However, the function of these ncRNAs is mainly unclear. Circular RNAs (circRNAs) include many miRNA-binding sites (miRNA response elements, MREs), which operate as miRNA sponges or competing endogenous RNAs (ceRNAs). The purpose of this study was to look at the role of circular RNAs (circRNAs) and microRNAs (miRNAs) in Alzheimer’s disease (AD) as possible biomarkers. The Gene Expression Omnibus (GEO) database was used to obtain an expression profile of Alzheimer’s disease patients (GSE5281, GSE122603, GSE97760, GSE150693, GSE1297, and GSE161435). Through preliminary data deletion, 163 genes with significant differences, 156 miRNAs with significant differences, and 153 circRNAs with significant differences were identified. Then, 10 key genes, led by *MAPT* and *AP2M1*, were identified by the mediation center algorithm, 34 miRNAs with obvious prognosis were identified by the cox regression model, and 16 key circRNAs were selected by the database. To develop competitive endogenous RNA (ceRNA) networks, hub circRNAs and mRNAs were used. Finally, GO analysis and clinical data verification of key genes were carried out. We discovered that a down-regulated circRNA (has_circ_002048) caused the increased expression of numerous miRNAs, which further inhibited the expression of a critical mRNA (*AP2M1*), leading to Alzheimer’s disease pathology. The findings of this work contribute to a better understanding of the circRNA-miRNA-mRNA regulating processes in Alzheimer’s disease. Furthermore, the ncRNAs found here might become novel biomarkers and potential targets for the development of Alzheimer’s drugs.

## Introduction

Alzheimer’s disease (AD) is a chronic neurological illness that worsens with time. Extracellular senile plaques created by β-amyloid protein deposition, neurofibrillary tangles formed by tau protein hyperphosphorylation, and neuronal loss with glial cell hyperplasia are the hallmark diseases. Despite substantial progress in identifying disease-related molecular and cellular processes over the last decade, the molecular mechanisms underlying the pathogenesis of AD remain largely unclear (Zádori et al., 2018), and none of the existing pharmaceutical therapies for AD can stop or reduce its progression (Association and #X, 2015). As a result, further research into the underlying disease processes is urgently needed to better understand AD and aid in the development of viable therapy options. Now, a comprehensive body of evidence shows that non-coding RNAs (ncRNAs), particularly microRNAs (miRNAs) and round RNAs (circRNAs), are being highlighted for involvement in pathophysiology associated with the promotion.

Non-coding RNA (NC) has no protein-coding activities but is an important regulator of many cellular processes (Barangi et al., 2019; Ueberham and Arendt, 2020). MicroRNAs (miRNAs) are tiny endogenous noncoding RNA molecules that suppress or mute post-transcriptional gene expression. Most miRNAs are highly conserved and play an important role in cell growth, differentiation, apoptosis, and post-transcriptional regulation (Natoli and Fernando, 2018). Previous research has found that numerous miRNAs, including miRNA-126a-3p, miRNA-1229, and miRNA-101a-3p, play a role in AD prevalence and pathogenesis. (Ghanbari et al., 2015; Lin et al., 2019; Xue et al., 2022), showing that miRNAs are strongly linked to the development of AD.

Prior research indicates that circRNAs may play important roles in cellular processes because of their abundance and evolutionary conservation. Several possible functions have been reported, including microRNA (miRNA) sponges. miRNA is also involved in gene expression regulation (Sanger et al., 1976; Capel et al., 1993; Hansen et al., 2013; Pamudurti et al., 2017), protein interaction intermediation, and transcription regulation of parental genes (Du et al., 2016). In addition, some circRNAs are associated with diseases by affecting genomic loci, suggesting that circRNAs may be involved in regulating related pathological processes (Li et al., 2017; Jiang et al., 2021). CircRNA promotes RNA translation and promotes small RNA assembly and stabilization. One of the characteristics of circRNA is that it is an evolutionarily conserved transcriptome with covalent connections at the 5 ‘and 3′ ends, derived from the backspacing of pre-mRNA backspacing and may be involved in gene regulation (Burd et al., 2010; Lukiw, 2013a; Li et al., 2015; Peifei et al., 2015). CircRNAs are much more stable than linear or non-coding mRNAs because they typically live hours to days or longer and lack 5′ and 3′ ends (Chen, 2016). Therefore, the biological function, stability, and specific expression of circRNAs may be different from other classes of RNAs, and the specific expression of some circRNAs has identified them as the best candidates for neurodegenerative disease biomarkers. Recent research suggests that the circRNA-associated ceRNA network is important in various disease processes, including Alzheimer’s (Frank-Kamenetskii, 2013). For example, Lukiw et al. demonstrated that circRNA-7 functions as a natural miRNA sponge for miRNAs-7 and regulates the expression of ubiquitin-conjugating enzyme *E2A* (*UBE2A*), and also showed that circRNAs are involved in the regulation of circulating miRNA genes in AD. Zhang et al., Found that the circRNA-associated ceRNA network in the mouse cerebral cortex is affected by mouse aging (Lukiw et al., 2016; Zhang et al., 2017). The underlying mechanisms and specific roles of circRNAs in AD pathology, however, remain obscure and remain to be investigated.

Here, we established a regulatory network of ncRNAs and mRNAs by integrating circRNAs, and mRNAs, and competing with them for co-binding miRNAs, in an attempt to gain a deeper understanding of the potential functions of the circRNA-miRNA-mRNA network in AD. These findings will help to better understand the underlying pathogenesis of AD and explore new potential biomarkers for the diagnosis and treatment of AD.

## Methodologies and materials

### Basic information about the database

All gene expression data sets used in this research were collected from the GEO database. (https://www.ncbi.nlm.nih.gov/geo/browse/). This database generated a total of 340 datasets on human Alzheimer’s disease. Three gene expression patterns (GSE5281, GSE97760, and GSE122063) were selected after careful examination. GSE5281 were among them based on the GPL570 (Affymetrix Human Genome U133 Plus 2.0 Array) platform, GSE97760 and GSE122063 were constructed on the Agilent GPL16699 platform (Agilent-039494 Sure Print G3 Human GE v2 8 × 60 K Microarray 039,381). All of the data were freely available online and the fact that these databases come from different platforms, different brain regions, different age groups, and different genders make it easier for us to identify genes that are generally significantly different. GSE5281Expression profile included 161 different age samples of 74 people without disease and 84 people with Alzheimer’s disease (∼55,000 transcripts). The data came from six different areas of the brain (entorhinal cortex, hippocampus, medial temporal gyrus, posterior cingulate, primary visual cortex, and superior frontal gyrus). The GSE97760 database analyzed the blood RNA of female patients with advanced AD and matched healthy controls, including 10 age-matched female healthy controls (age 72.1 ± 13.1 years) and 9 advanced AD patients (age 79.3 ± 12.3 years). GSE122063 includes 56 disease (AD) and 44 non demented controls (controls) from various age stages (60–91). AD gene expression profiles were obtained from frontal and temporal cortices of autopsy hemispheres collected from the University of Michigan, United States (Liang et al., 2007; Naughton et al., 2015; McKay et al., 2019). Download GSE150693 data from the GEO database, including 197 different age (59–94) serum mild cognitive impairment (MCI) miRNA samples, of which mild cognitive impairment (MCI) is a clinical precursor to Alzheimer’s disease (AD) (114 MCI non-transformers: MCI-NC and 83 MCI convert: MCI-C) (Shigemizu et al., 2020). We attempt to identify aberrant circRNAs in the peripheral whole blood of AD patients by circular RNA microarray using the GSE161435 database, including three AD patients and three control individuals (Liu et al., 2020). For GSE1297 data, gene expression analysis of the hippocampus from nine control subjects and 22 AD subjects of varying severity was performed on 31 separate microarrays. Some clinical information is also included such as MiniMental Status Examination (MMSE) and neurofibrillary tangle examination (NFT) were also included (Blalock et al., 2004).

### Data processing of differentially expressed (DE) ncRNAs and DEGs

Data were processed using DEG’s GEO2R online analysis tool (https://www.ncbi.nlm.nih.gov/geo/geo2r/), and adjusted *p*-values and |logFC| were calculated. For the gene expression profiles of GSE5281 and GSE97760, Log2-FoldChange (FC)|>1.0 and an adjusted *p*-value <0.05 were used as cutoff criteria, and the adjusted *p* < 0.05 and |logFC|>0.5 the gene expression profiles of GSE122063 were treated as DEGs. Differential miRNAs were screened and selected from GSE150693 by the ‘limma’ package in the ‘R' language based on the criteria of *p* < 0.05 and FC > 1. Differential circRNAs were first screened in GSE161435 based on the criteria of *p* < 0.05 and FC > 1.5.

### KEGG pathway and GO analysis of DEGs

The KEGG database is widely used and stores large amounts of data on genomes, biological pathways, diseases, and medicines. GO analysis is a common method for large-scale functional enrichment studies; gene functions can be divided into the biological process (BP), cellular component (CC), and molecular function (MF). KEGG pathway enrichment analysis of DEGs in this study was performed using the Database Annotation, Visualization, and Integrated Discovery (DAVID) tool (https://david.ncifcrf.gov/). For KEGG pathway analysis, *p* ≤ 0.01 and count≥4 were considered sufficient and analysis results were visualized using the online analysis tool Sangerbox (http://vip.sangerbox.com/home.html). To better analyze the function of the DEGs gene, in this study, biological processes (BP), cellular components (CC) molecular functions (MF), and molecular functions (MF) are annotated, analyzed, and visualized using R language “cluster profile”, “GGploT2 “and” enrichPlot “packages.

### Hub genes and PPI network constructs

The Search Tool for the Retrieval of Interacting Genes (STRING) database (http://string-db.org/) is designed to analyze the PPI information. To evaluate the potential PPI relationship, the DEGs identified previously were mapped to the STRING database. The PPI pairs were extracted with a combined score = 0.4. Subsequently, the PPI network was visualized by Cytoscape3.9 software (http://www.cytoscape.org/).To better search for proteins related to Tau protein, the algorithm Between Centrality was adopted in this study to find the important nodes (Formulas 1 and 2). CytoHubba, a plugin in Cytoscape, calculates the nodes in the protein network that transmit information efficiently. In our study, the top 10 genes were identified as pivotal.

$$C_{B}\left(N_{i}\right)=\overset{g}{\underset{k,j=1}{\sum }}sd\left(k,i,j\right),\left(k\neq j\right)$$ (1)

Where sd (k, i, j) means that the shortest path from k to j passes through i, that is, i am on the shortest path from k to j.

$$C_{B}^{\prime }\left(N_{i}\right)=\frac{C_{B}\left(N_{i}\right)}{\overset{g}{\underset{k,j=1}{\sum }}\left(k,j\right)},\left(k\neq j\right)$$ (2)

The formula can be standardized to obtain (2), where the denominator represents the number of paths between two points in the figure, that is, the number of all paths.

### Delete hub miRNA and circRNA

Through the ‘R' language ‘limma’ package, based on *p* < 0.05 and FC > 1 criterion, a total of 156 miRNAs were identified from GSE150693, 141 of which were upregulated and 15 downregulated. Then in this study, to integrate survival time, AD transformation status, and 35 feature data, we used the ‘R' software package ‘survival’ and assessed the prognostic significance of these features in 197 samples by the cox method. Overall prognosis difference was significant (LOGtest = 0.028469442826945, SCtest = 0.041660198345316,WALdtest = 0.04901065748048,C-index: 0.727997187390132).

Then based on the criteria of KM-Pvalue<0.01, the Top 34 miRNA with the most obvious prognosis were selected as research objects. According to criteria *p* < 0.05 and FC > 1.5, difference circRNA was preliminarily screened in GSE161435, including 18 upregulated and 87 downregulated circRNA. Then we selected 16 important circRNAs from the ENCORE database.

### Prediction of miRNAs and analysis of competing endogenous RNA network

We used ENCORE to predict circRNA and mRNA target miRNAs. The ceRNA network was constructed using Cytoscape, based on the function of circRNA, miRNA, and mRNA sponge.

### GSEA and clinical information validation

For Gene Set Enrichment Analysis (GSEA), we obtained data from GSEA (DOI: 10.1073/pans. 0,506,580,102, http://software.broadinstitute.org/gsea/index.jsp) website for the GSEA software (version 3.0). The specimens were divided into highly expressed groups (>=50%) and low expression groups (<50%) according to the expression level of genes, and obtained from Molecular Signatures The Database (DOI:10.1093/bioinformatics/btr260,http://www.gsea-msigdb.org/gsea/downloads.jsp) to download a subset of“c3.mir.v7.4.symbols.gmt”.To evaluate related pathways and molecular mechanisms, based on gene expression profile and phenotype grouping, the minimum gene set was 5, the maximum gene set was 5,000, 1,000 resampling, *p*-value of <0.05, and an FDR of <0.25 were considered statistically significant. To verify the binding relationship of miRNAs to target genes by GSEA.

In the GSE1297 database, the key gene data were normalized, and then Correlation analysis with clinical information mmse, NFT, and braak.

## Result

### Identification of DEGs

**FIGURE 1:**
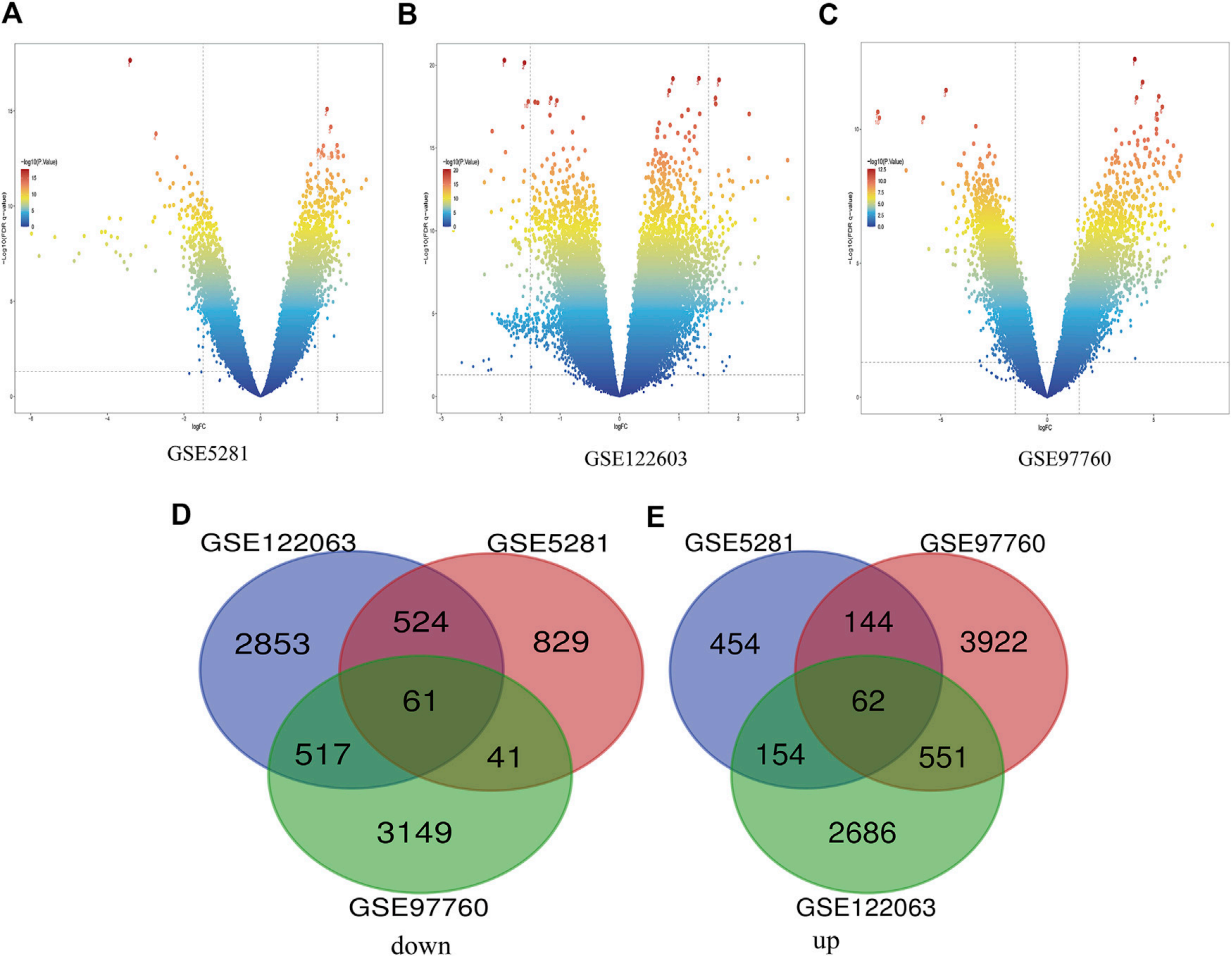
Differential expression of AD genes **(A)** Volcano plot of GSE5281 differential genes, **(B)** Volcano plot of differentially expressed genes in GSE122603, **(C)** The volcano map of GSE97760’s expression levels in AD vs. control. blue dots represent genes that are not altered. Other colors represent Represents differentially expressed genes, where the closer the color is to red, the greater the multiplicity of differences. **(D)** and **(E)** DEG (differentially expressed gene) Venn diagram common to the three GEO datasets, **(D)** Downregulated genes, **(E)** Upregulated genes.

In this study, three gene databases (GSE5281, GSE97760, and GSE122063) were chosen for analysis. Based on the criteria of |logFC|>1 and adjusted *p* < 0.05, a total of 2,269 DEGs were identified from GSE5281, including 1,455 down-regulated genes and 814 up-regulated genes. GSE97760 gene array identified 8,447 DEGs; 4,679 genes were upregulated and 3,768 genes were downregulated. And based on the adjusted *p* < 0.05 and |logFC|>0.5 criteria, 3,955 down-regulated genes and 3,453 up-regulated genes were identified from GSE122063, totaling 7,408 DEGs. Identification of all DEGs was based on a comparison of AD samples with normal ones. Then, take the intersection of all DEGs of the three data to make a Venn diagram, (Figures 1D,E), and the volcano plots of the three gene expression matrices are shown in Figure 1. Eventually, a total of 123 DEGs with significant differences were identified, 62 of which were significantly down-regulated and 61 of which were up-regulated.

### Enrichment analysis and pathway analysis of differential genes

**FIGURE 2:**
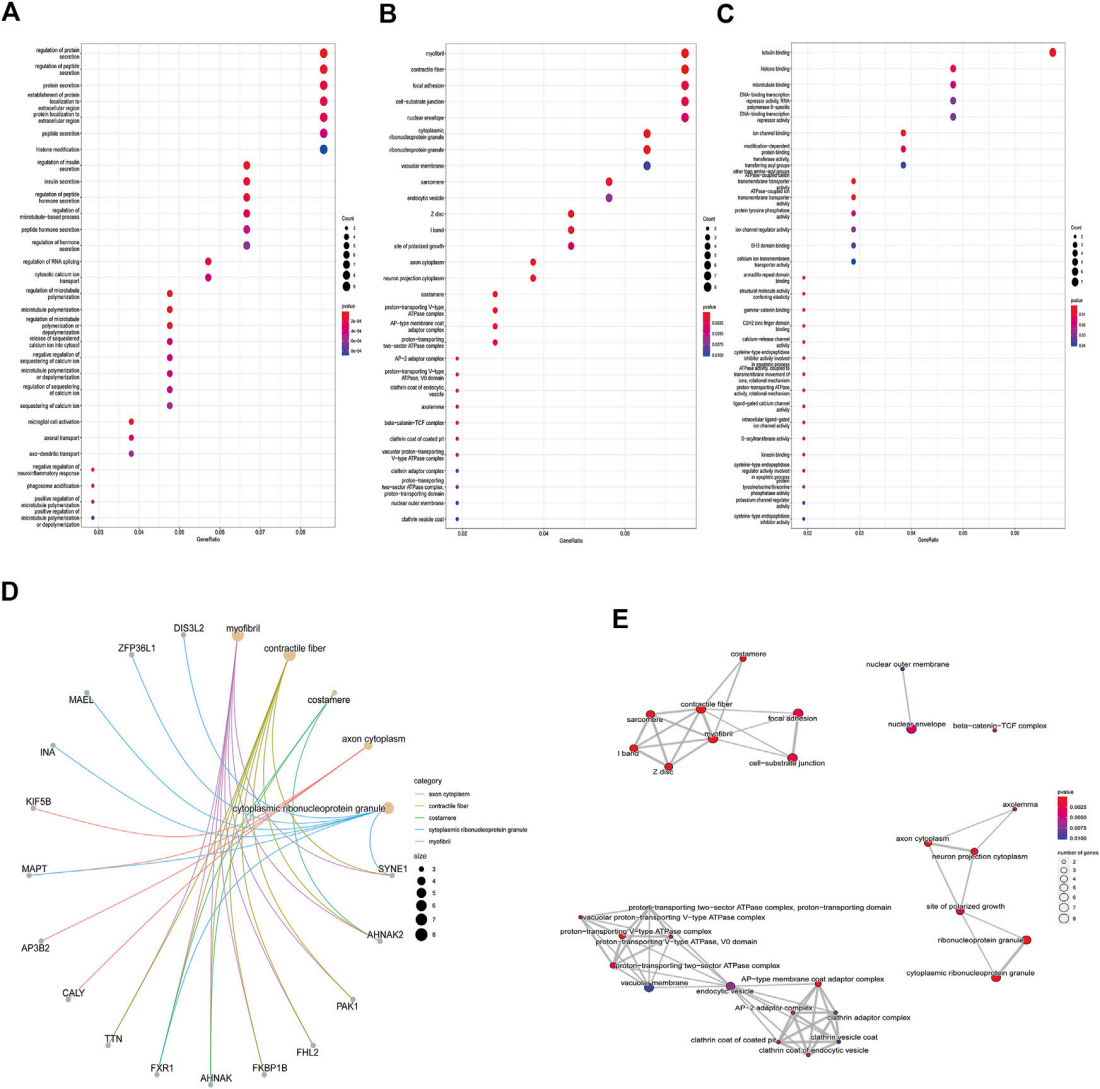
GO analysis results of DEGs. Panel **(A)**. GO biological process enrichment results; panel **(B)**. GO cell component enrichment results. Panel **(C)**. GO molecular function enrichment results. Panel **(A–C)**. Each bubble represents the fitted *p*-value: the redder the color, the higher the enrichment. Panel **(D)** and **(E)**. The genes contained in the more enriched category, and the relationship between the classes in the GO cell component.

Enrichment analyses of KEGG pathway enrichment and GO functional enrichment analysis of DEGs by DEGs was performed using DAVID And R package “cluster profile” (Table 1, 2). GO terms enriched in the database were split into BP, MF, and CC ontologies. According to GO analysis results, DEGs were mainly enriched in BPs, including regulation of protein secretion, regulation of peptide secretion, downregulation of neuroinflammatory response, regulation of RNA splicing, transcription regulation from RNA polymerase II promoter, differentiation of muscle cells, and signal release. MF analysis showed that the DEGs were protein binding, tubulin binding, histone binding, DNA−binding transcription repressor activity, RNA polymerase II−specific and ion channel binding. For the cell component, the DEGs are myofibril, perinuclear region of cytoplasm, cell−substrate junction, cytosol, cytoplasm, tubulin binding nuclear envelope, ribonucleoprotein granule, and focal adhesion (Figure 2). Through KEGG pathway analysis, DEGs were mainly enriched in *Salmonella* infection, Epithelial cell signaling in *Helicobacter pylori* infection, MAPK signaling pathway, and Synaptic vesicle cycle, neuron projection cytoplasm.

**TABLE 1:** Significantly enriched GO terms of DEGs.

Category	Term	Description	*p*-value	Count
BP term	GO:0050708	regulation of protein secretion	2.59E-05	9
BP term	GO:0002791	regulation of peptide secretion	5.60E-05	9
BP term	GO:0009306	protein secretion	0.000210732	9
BP term	GO:0035592	establishment of protein localization to the extracellular region	0.000215028	9
BP term	GO:0071692	protein localization to the extracellular region	0.000247183	9
BP term	GO:0002790	peptide secretion	0.000403691	9
BP term	GO:0016570	histone modification	0.000891138	9
BP term	GO:0016569	covalent chromatin modification	0.001088715	9
BP term	GO:0042692	muscle cell differentiation	0.000942916	8
BP term	GO:0023061	signal release	0.005053118	8
BP term	GO:0008380	RNA splicing	0.005441099	8
CC term	GO:0030016	myofibril	3.26E-05	8
CC term	GO:0043292	contractile fiber	4.06E-05	8
CC term	GO:0005925	focal adhesion	0.002066786	8
CC term	GO:0030055	cell-substrate junction	0.002291647	8
CC term	GO:0005635	nuclear envelope	0.003919523	8
MF term	GO:0015631	tubulin binding	0.004981899	7
MF term	GO:0044325	ion channel binding	0.006085711	4
MF term	GO:0019829	ATPase-coupled cation transmembrane transporter activity	0.001982463	3
MF term	GO:0042625	ATPase-coupled ion transmembrane transporter activity	0.002253707	3

Abbreviations: GO, Gene Ontology; KEGG, Kyoto Encyclopedia of Genes and Genomes.

**TABLE 2:** KEGG pathways of DEGs.

Category	Term	Description	*p*-value	Count
KEGG_PATHWAY	hsa05132	*Salmonella* infection	0.007079	7
KEGG_PATHWAY	hsa05120	Epithelial cell signaling in *Helicobacter pylori* infection	0.012308	4
KEGG_PATHWAY	hsa04010	MAPK signaling pathway	0.015277	7
KEGG_PATHWAY	hsa04721	Synaptic vesicle cycle	0.016457	4

### PPI network construction and core gene screening

**FIGURE 3:**
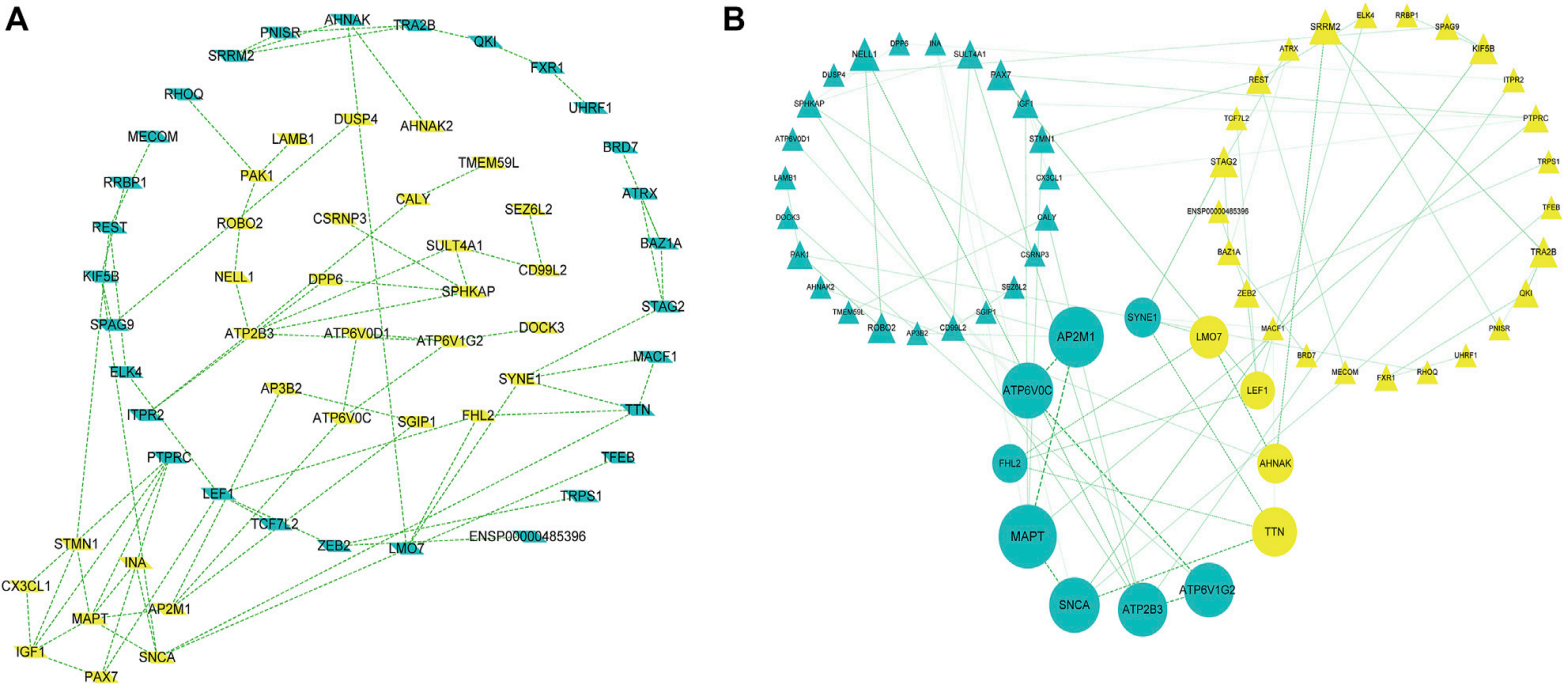
A protein-protein interaction network is built with the differentially expressed genes **(A)** Green diamond nodes represent down-regulated genes and yellow diamond nodes represent up-regulated genes. **(B)** Interaction networks are curated using Between Centrality algorithm, Nodes in green triangles represent downregulated genes, and nodes in yellow triangles represent upregulated genes. Small balls represent the final 10 hub genes, and the larger the ball, the higher the score.

PPI network construction and core gene screening Prediction of protein interactions between DEGs using the STRING database. A total of 119 nodes and 78 edges are involved in filtering through the PPI network (Figure 3A). In the PPI network, between Centrality algorithm is used to measure the extent to which a node can become an “intermediary”, that is, the extent to which it controls other genes, and the first ten genes are selected as research objects, as presented in Figure 3B.

The results showed that encodes of the microtubule-associated protein tau (*MAPT* were the most outstanding gene with a score = 1820, followed by encoded as a subunit of the heterotetrameric coat assembly protein complex2 (*AP2*) (*AP2M1*;score = 1,672), encodes a component of vacuolar ATPase (V-ATPase) (*ATP6V0C*; score = 1,476). *SNCA* (Synuclein Alpha) is a Protein Coding gene. It may serve to integrate presynaptic signaling and membrane trafficking (*SNCA*; score = 1,443.7), the Catalytic subunit of the peripheral V1 complex of vacuolar ATPase (V-ATPase) (*ATP6V1G2*; score = 1,406), And the clinical information is verified in the database GSE1297. The top 10 hub genes are shown in Table 3.

**TABLE 3:** Hub genes.

Rank	Name	Score
1	MAPT	1820
2	AP2M1	1,672
3	ATP6V0C	1,476
4	SNCA	1,443.7
5	ATP6V1G2	1,406
6	ATP2B3	1,386
7	TTN	1,160.333
8	LMO7	862.6667
9	SYNE1	741.5
10	AHNAK	740

### Analysis of hub miRNAs and circRNAs

**FIGURE 4:**
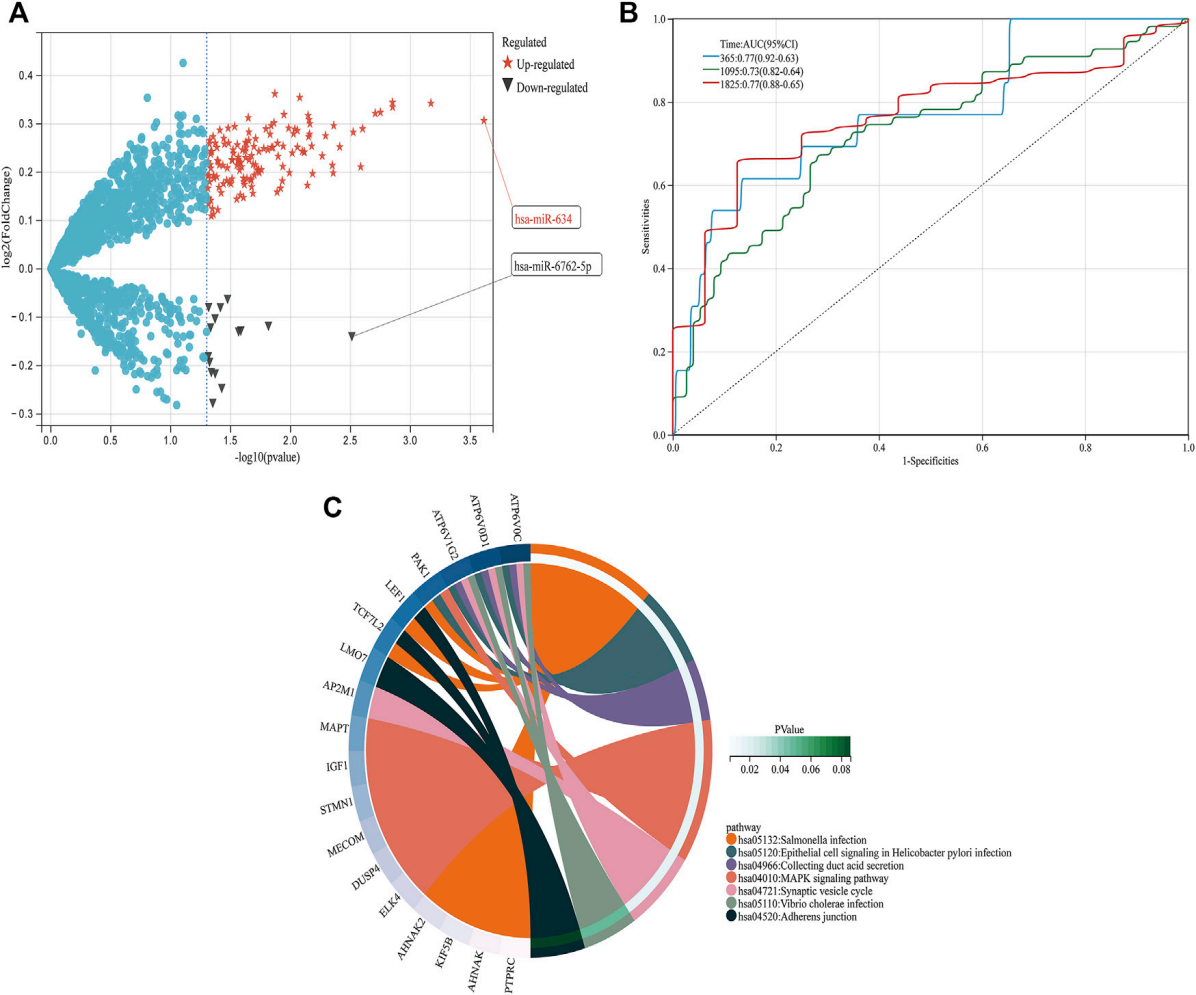
DEGs pathway, differential expression of miRNAs, and prognosis **(A)** In the volcano graph of differentially expressed circRNAs, red five-pointed stars are up-regulated and black triangles are down-regulated miRNAs. **(B)** ROC analyses for the 365, 1,095, and 1825 time points were performed, **(C)** KEGG pathway diagrams of DEGs.

**FIGURE 5:**
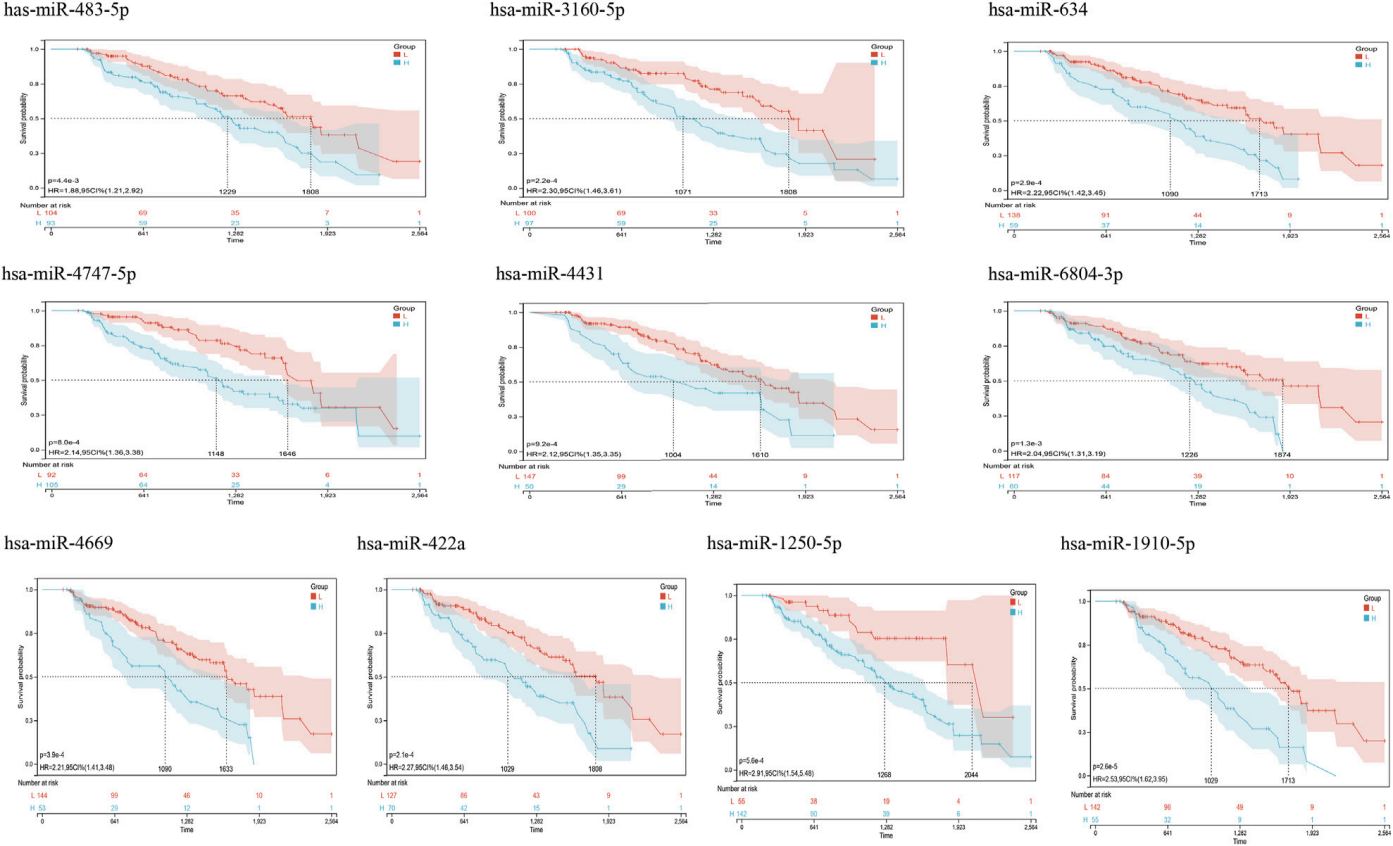
Kaplan Meier curves for survival without conversion to AD generated by the prediction models. We evaluated the prognostic significance of these features in 197 samples by integrating data on survival time, transformation status of AD, and 35 features using the Cox method to calculate a prognostic index for each subject. We divided the sample of the discovery cohort into a high (blue) risk group and a low (red) risk group according to the prognostic index. Survival differences without MCI to AD conversion were compared by using the minimum *p*-value of the log-rank test to detect the optimal cutoff. In the graph are the top 10 miRNAs with the smallest *p*-value of detection.

**FIGURE 6:**
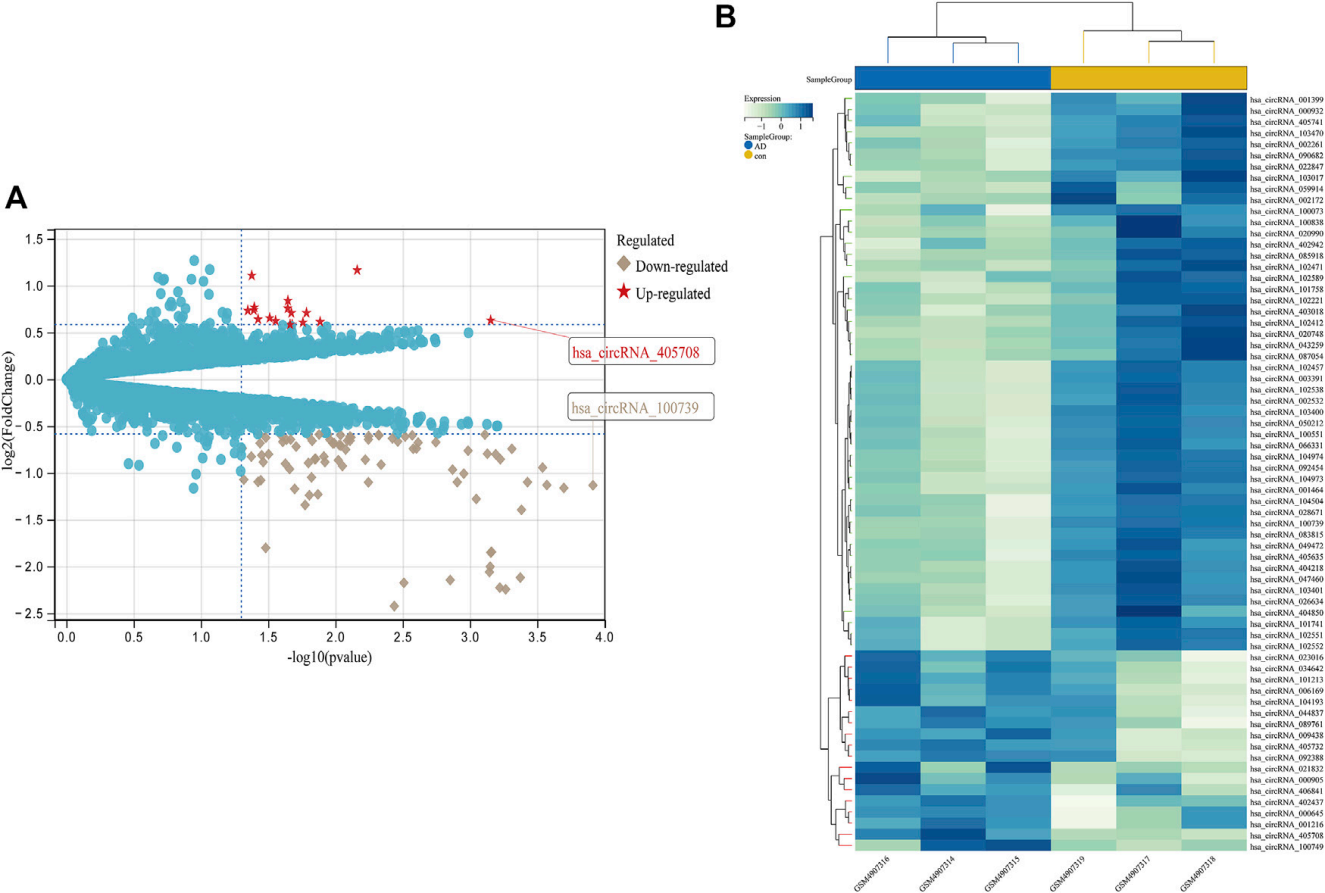
Differential expression diagram of circRNAs, **(A)** differential expression volcano plot of circRNAs, with red pentangles being upregulated and gray diamonds being downregulated circRNAs, **(B)** Heatmap of differentially expressed circRNAs.

From GSE150693, a total of 156 differential miRNAs were identified by the “limma” package of the “R" language, 141 of which were upregulated and 15 downregulated. Secondly, in this study, to explore the prognostic value of potential central miRNA, we used the “R" package “Survival Rate” to integrate survival, AD transition status data, and 35 characteristics, and evaluated the prognostic significance of these characteristics in 197 samples by the cox method (Figures 4A,B). Overall prognosis difference was significant (LOGtest = 0.028469442826945, SCtest = 0.041660198345316, WALdtest = 0.04901065748048),C-index: 0.727997187390132).Then based on the criteria of KM-Pvalue<0.01, the Top 34 miRNA with the most obvious prognosis were selected as research objects. As shown in Figure 5, we showed the AD conversion status of the top 10 miRNAs. Basing on the criterion of *p* < 0.05 and FC > 1.5, a total of 105 circRNAs were found from 13,617 circRNAs in GSE161435 using the ‘R' language ‘limma’ package. Then, utilizing the carcass database, circRNAs with obvious records and biological verification were identified, and 16 hub circRNAs were constructed, as shown in Figure 6.

### Construction of the ceRNA network

**FIGURE 7:**
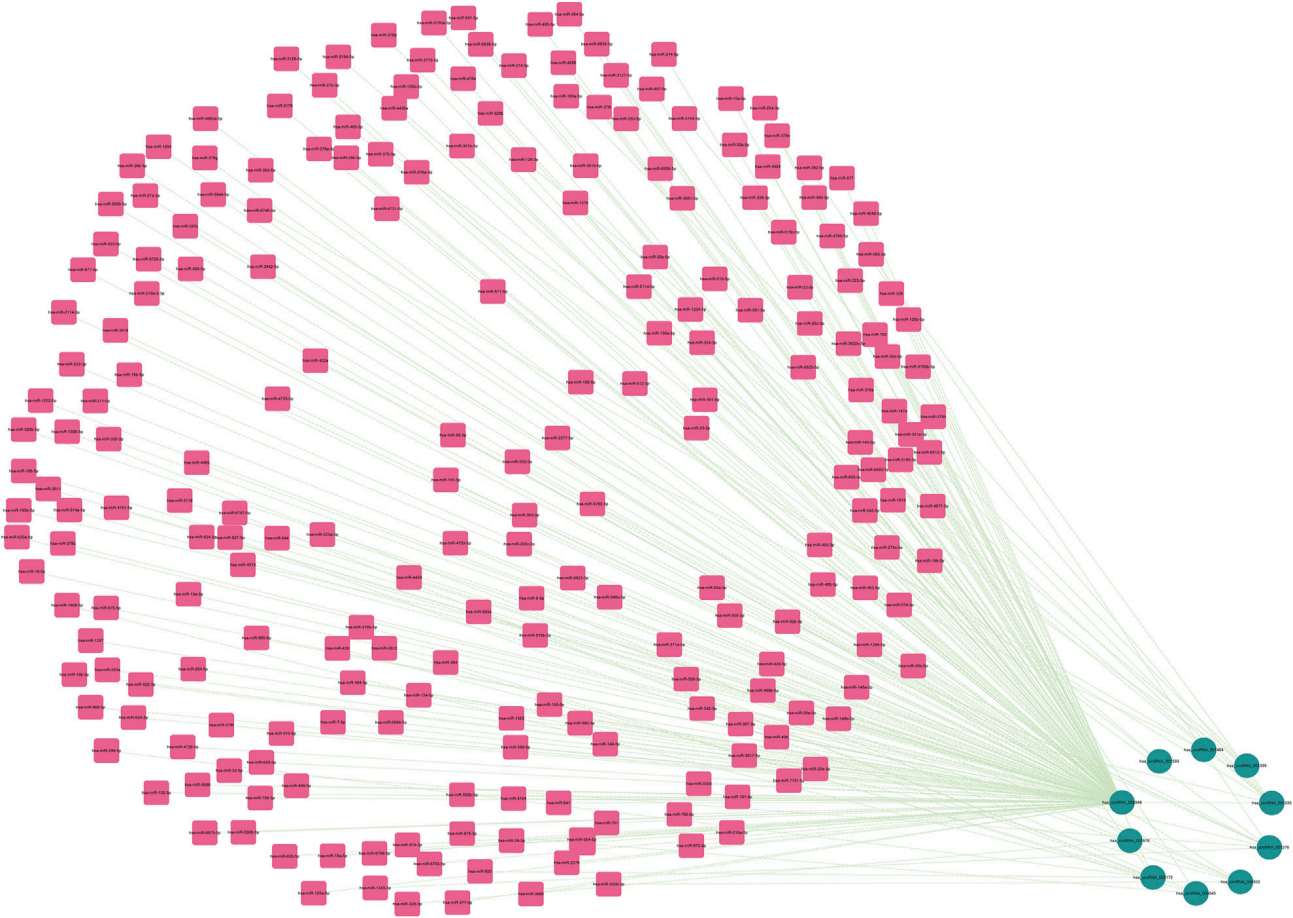
The miRNAs to which 10 key circRNAs bind are predicted. In the network, thin lines represent sequence matches, green balls represent circRNAs and red rectangles represent miRNA.

**FIGURE 8:**
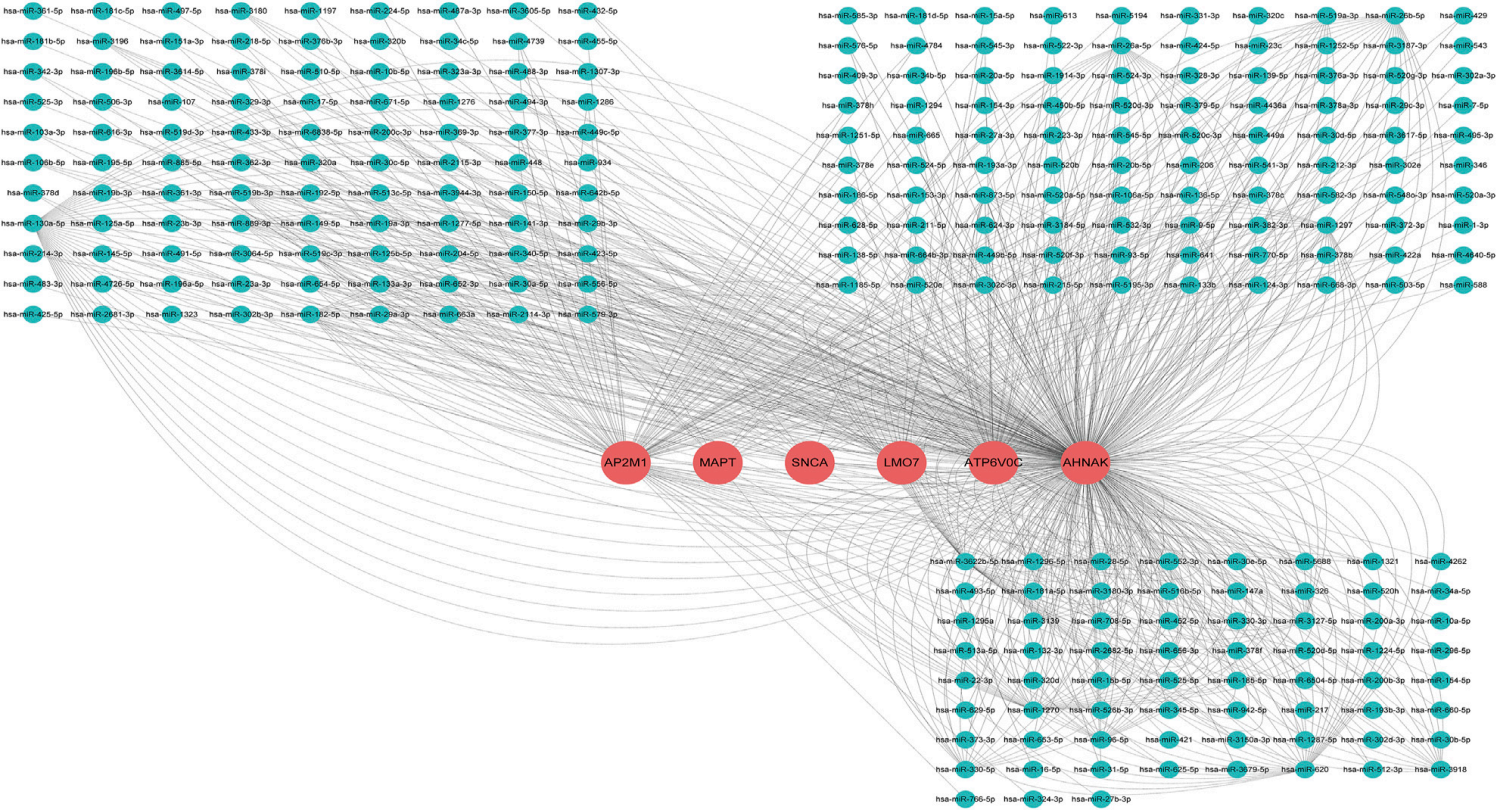
miRNA network targeting the hub genes of the final screen, in the network, thin lines represent targeting relationships, red balls represent mRNAs and green balls represent miRNAs.

**FIGURE 9:**
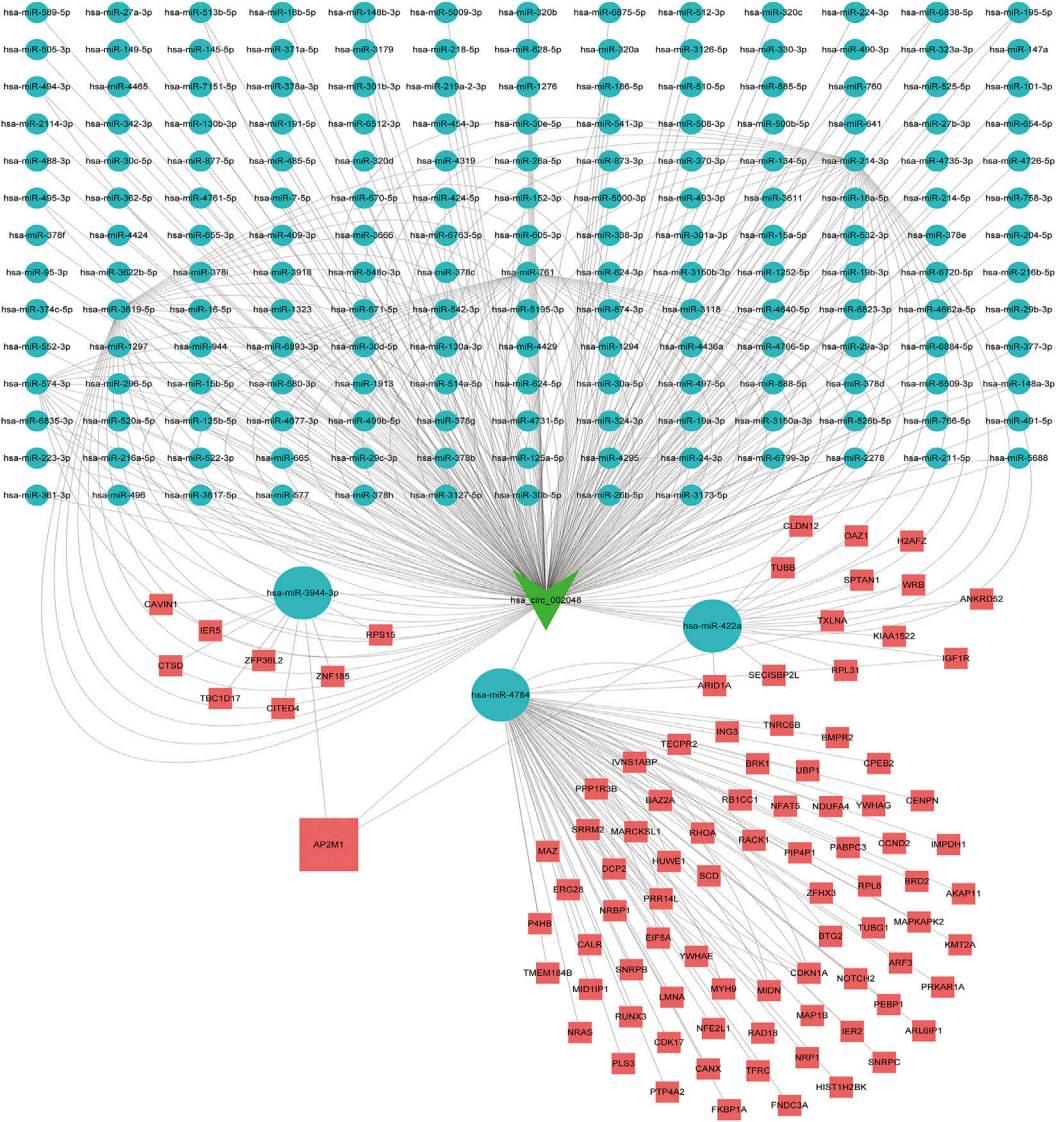
Competitive endogenous RNA networks. Competing endogenous RNA networks were constructed based on the circRNA/miRNA interaction and the miRNA/mRNA interaction. Reseda V-type represents circRNAs, green balls represent miRNAs and red rectangles represent mRNAs.

As is well known, circRNAs function as sponges of miRNAs to regulate mRNA function. Thus, we used the ENCORI database to predict the binding relationship between differentially expressed circRNAs and miRNAs. The ceRNAnetwork hypothesis states that RNAs from competing for endogenous RNAs (ceRNAs) compete for the same miRNA response elements to affect one another. Furthermore, We intersected the selected miRNAs with 34 miRNAs in the GSE150693 database to identify common miRNAs and establish key circRNA-miRNA pairs. Through the screen out of the ENCORI database, only 10 of the 16 circRNAs were eligible, and the miRNAs that bound to them were predicted (Figure 7). The results showed that the binding of circRNA and miRNA is not one-to-one, specific circRNA can interact with multiple miRNAs, and circRNA may affect AD progression by regulating various miRNAs. We used the ENCORI database, which includes miRNA-mRNA (CLIP) > 2,500,000 interactions to predict the target mRNAs of the selected miRNAs. To improve the accuracy of prediction, “AgoExpNum” must be greater than or equal to 10 in the database, and A total of 6 hub genes and their associated miRNAs were screened out (Figure 8). We constructed the final circRNA-miRNA-mRNA network map using Cytoscape3.9 software, as shown in Figure 9.

## Discussion

Alzheimer’s disease (AD), the most common neurodegenerative disorder, is a progressive neurologic disorder with insidious onset and is the primary cause of dementia. In AD, two hallmarks are intracellular neurofibrillary tangles of hyperphosphorylated tau and accumulation of beta-amyloid plaques. (Mandelkow, 1992; Citron et al., 2001). β-amyloid and tau proteins have been the focus of research on Alzheimer’s disease and drug development for decades. The majority of therapeutic studies targeting amyloid, Although the effectiveness of most tau-targeted drugs has not been fully evaluated, this has led to a greater understanding of the complexities of AD and attempts to explore new drug mechanisms. The onset and course of Alzheimer’s disease are influenced by very complicated interactions between hereditary, epigenetic, and environmental variables. Therefore, the identification of more appropriate network regulation of the circRNA–miRNA–mRNA axis is essential to guide the individualized treatment of AD.

Luckily, we discovered that in GO enrichment analysis, DEGs were mainly enriched in tubulin binding, ion channel binding, nuclear envelope, regulation of protein secretion, signal release, protein localization to the extracellular region, and so on. Among them, *AP2M1* is mainly enriched in ion channel binding and inner cytocapsula. They may have a potential link with AD (Schmitt, 2005; Kimura and Yanagisawa, 2018). More interestingly, in KEGG pathway analysis, *AP2M1* was predominantly enrichment in the synaptic vesicle cycle pathway. Neurons are the most fundamental structural and functional.

Unit of the nervous system. The Synapse is an asymmetric structure that is essential for neuronal function. The chemical transmission mode of the synapse is realized through neurotransmitters and electrical processes. Based on vesicle transport, the abnormal information transmission process in the synapse can lead to a series of related diseases (Wei et al., 2022). The Synapse is the basic structural and functional component for neural communication in the brain. Neurotransmitter release is mediated by exocytosis of synaptic vesicles at the presynaptic active zone of nerve terminals (Sudhof, 2004). The presynaptic terminal is the structural and functionally essential area that initiates communication and maintains the continuous functional neural information flow. Synaptic alteration is considered to be central to neural disease processes. In particular, the alteration of the structural and functional phenotype of the presynaptic terminal is highly significant evidence for neural diseases, including Alzheimer’s disease (AD), Parkinson’s disease (PD), Amyotrophic lateral sclerosis (*ALS*), and Huntington’s disease (HD) (Bae and Kim, 2017; Ovsepian et al., 2018). The synaptic vesicle cycle (SVC) holds center stage in the biology of presynaptic terminals. Through recurrent exocytosis and endocytosis, it facilitates a sequence of events that enable chemical neurotransmission between functionally related neurons. In AD, SVC is both the primary site of amyloid β production and toxicity (Bonnycastle et al., 2021). It has also been shown that *ATP6V1A* downregulation is involved in AD through a synaptic vesicle cycle (Zhou et al., 2021).

In the whole development of AD, the circRNA-miRNA-mRNA regulatory network plays a crucial role (Ma et al., 2019). For example, circular RNA (circRNA) ciRS-7 in Alzheimer’s disease (AD) targets the trafficking of miRNA-7 and promotes overexpression of the ubiquitin-conjugating enzyme (*UBE2A*) defective in the expression of epidermal growth factor receptors (*EGFR*), thereby affecting the pathology of AD (Lukiw et al.). The researchers also found a potential regulatory role of circRNA/ceRNA in Alzheimer’s disease in the *cGMP-PKG* signaling pathway (Zhang et al., 2021). The candidate functions of circRNAs/miRNAs as novel biomarkers and cancer therapeutic targets are of particular interest, and similarly, the study of circRNA and miRNA expression profiles by microarrays and further evaluation of candidate ncRNAs as possible candidates for Alzheimer’s disease (AD) biomarker potential (Li et al., 2020). This study aims to search for circRNAs, miRNAs, and mRNAs in different AD and will establish a circRNA-miRNA-mRNA endogenous competitive regulatory network to understand their roles in controlling downstream regulatory elements and their processes.

In recent years, research data have shown that circRNAs are involved in a series of biological processes such as cell proliferation, differentiation, gene expression, and immune response regulation. Many dysregulated circRNAs have been reported previously in many diseases, including AD (Meng et al., 2017; Ghafouri-Fard et al., 2022). Earlier studies have shown that the circRNA-mediated “miRNA sponge system” is defective, and inducing environmental upregulation of specific miRNAs may help explain the widely observed, general, and progressive downregulation of gene expression, which may explain the downregulation of many genes in the AD brain (Loring et al., 2001), Defects in ciRS-7 and “sponge activity”, for example, may increase ambient miRNA-7 levels in AD-affected brain cells, ultimately leading to downregulation of messenger RNAs (mRNAs) that selectively bind miRNA-7 (Lukiw, 2013b). Therefore, we tried to select the key circRNAs in this study by studying their corresponding miRNAs and tried to build a circRNA-miRNA network. In our work, we intersected 10 candidate circRNAs targeting 430 targeted miRNAs and 34 significant miRNAs from the GSE150693 database. has-miR-422a, has-miR-4784, and the-miR-3944-3p were found to be miRNAs regulated by hsa_circ_002048.

miRNAs are a kind of tiny, non-coding RNA that only has 20–22 nucleotides (Bartel, 2004). miRNAs control more than 60% of protein expression and are linked to a variety of neurodegenerative disorders (Juźwik et al., 2019; Ammal Kaidery et al., 2021). Accumulating data suggests that the deregulation of particular miRNAs engaged in critical regulatory genes is related to AD etiology and progression (Silvestro et al., 2019), hence, miRNA-mediated control presents a novel treatment target (Maffioletti et al., 2014). As miRNAs are persistent in biological fluids such as plasma, serum, and CSF, analyzing them in bodily fluids is a very easy, safe, and noninvasive technique (Backes et al., 2016; Kalogianni et al., 2018). Analyzing miRNA in a patient’s body fluids seems beneficial, and circulating miRNA seems to be the most reliable biomarker for AD diagnosis. (Fransquet and Ryan, 2018; Angelucci et al., 2019; Zhao et al., 2019). Existing studies attempt to analyze miRNA in the body fluids of AD patients, to use circulating miRNA from blood as potential biomarkers for AD pathology, The miRNA in our study was also extracted from the blood. (Pang et al., 2017; Fransquet et al., 2018; Zhao et al., 2018; Angelucci et al., 2019).

**FIGURE 10:**
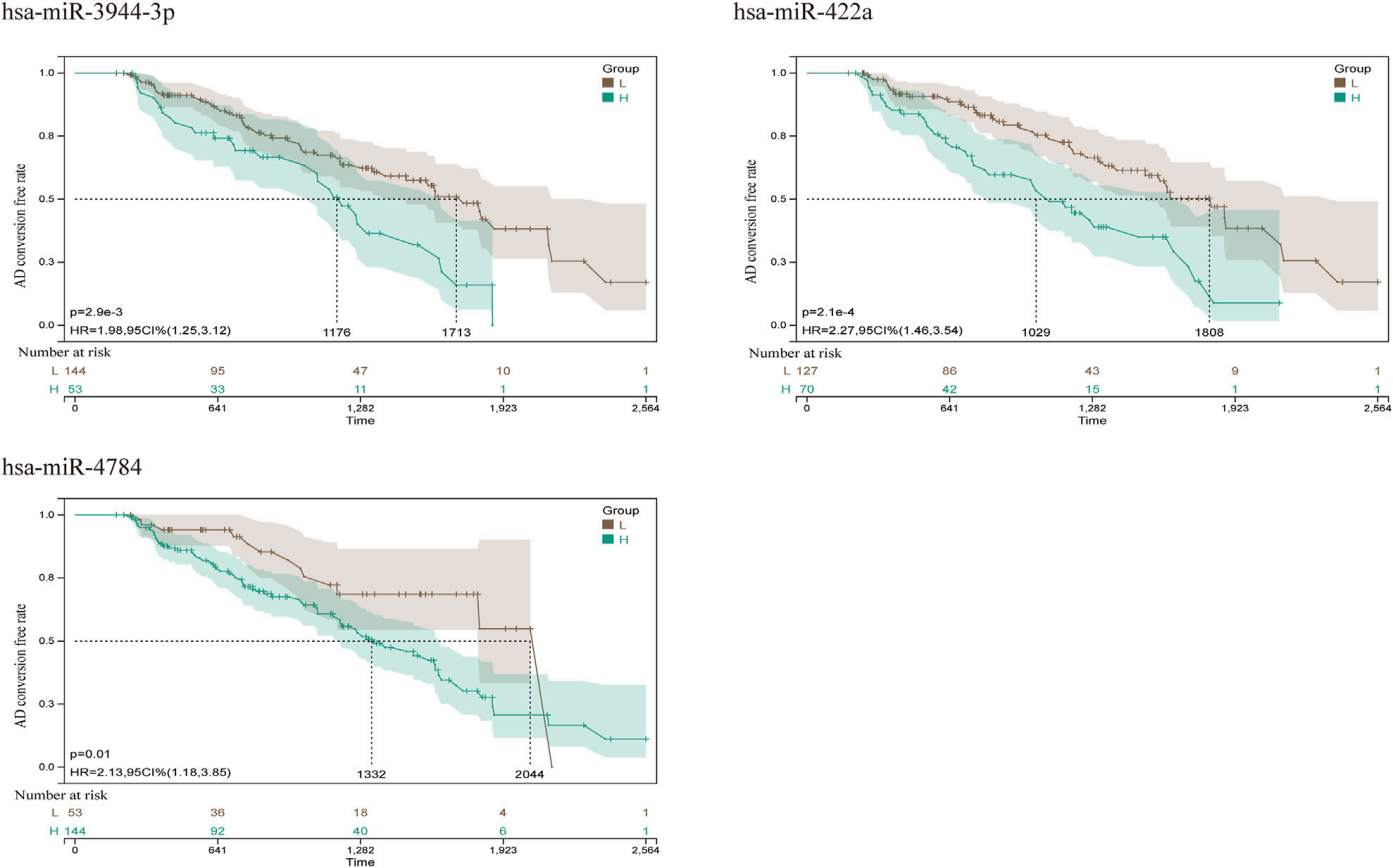
AD conversion curve of hsa-miR-422a, hsa-miR-4784, and hsa-miR-3944-3p. By integrating the data of survival time, AD transformation status, and 35 features, the prognostic significance of these features in 197 samples was evaluated using the cox method, and the prognostic index of each subject was calculated. According to the prognostic index, we divided the samples of the cohort into a high (green) risk group and a low (gray) risk group. The best cut-off value was detected by using the minimum *p*-value of the log-rank test to compare the difference in survival without MCI to AD conversion.

**FIGURE 11:**
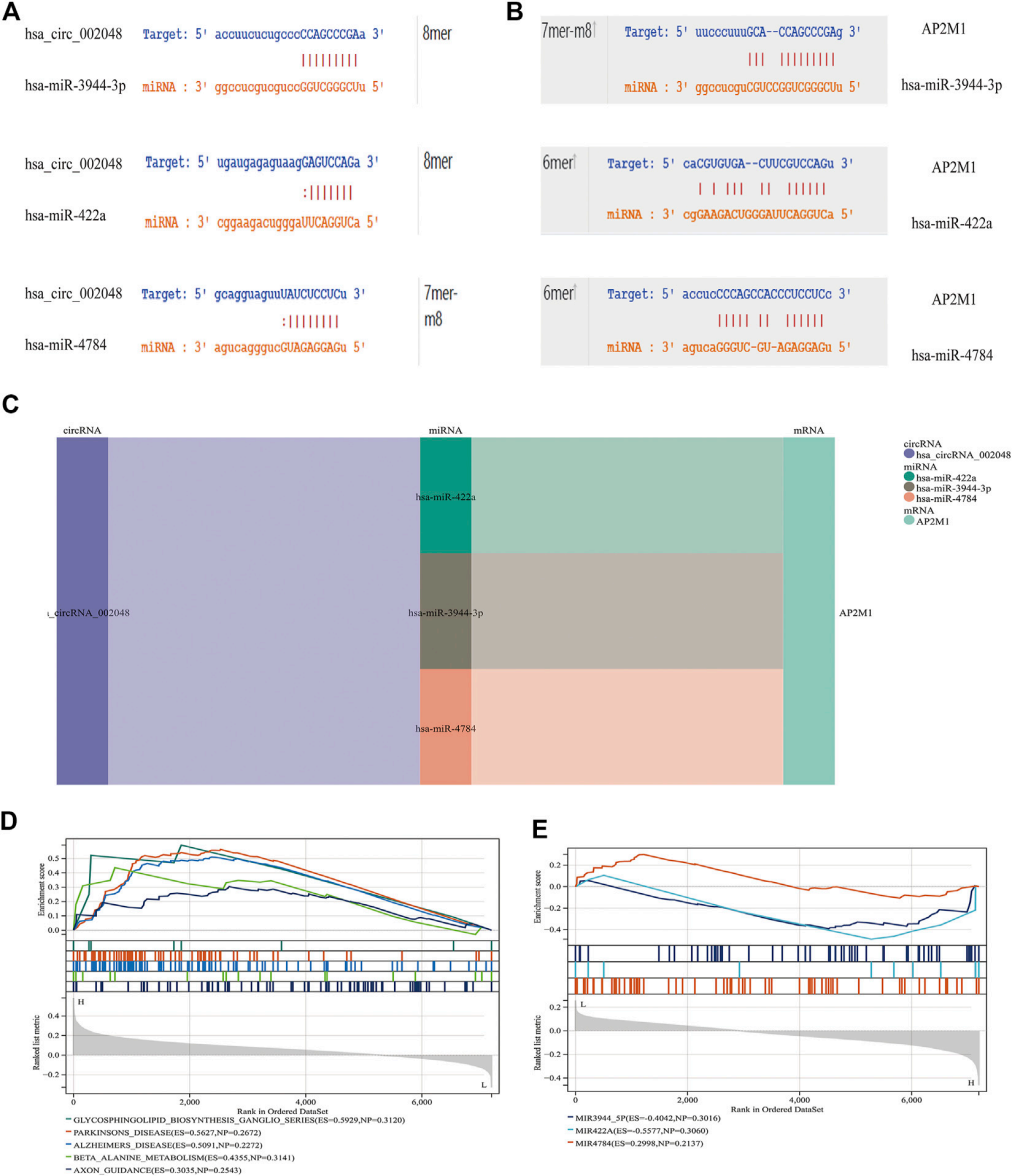
CircRNA/miRNA, miRNA/mRNA interaction **(A)** miRNAs interacting with hsa_circ_002048. **(B)** miRNAs that target *AP2M1* are shown. 6mer: Bases 3-8 are perfectly matched; 7mer-m8: Base 1 is not A, and bases 2-8 are perfectly matched; 8mer: Base 1 is A, and bases 2-8 are perfectly matched **(C)** Delineating the circRNA–miRNA–mRNA network with a Sankey diagram **(D)** In KEGG gene sets, GSEA results show that *AP2M1* is mainly enriched in glycosphingolipid biosynthetic ganglion series, Parkinson’s disease, Alzheimer’s disease, β- Alanine metabolism, and axonal guidance mechanism. **(E)** GSEA was used to assess the relevant pathways and molecular mechanisms, suggesting that miRNAs and their target gene AP2M1 may be co-enriched in the same molecular machinery (in all microRNA targets).

To further explore the blood-related miRNAs, which could act as biomarkers in AD, we used the ‘R' software package ‘survival’ to integrate the data of survival time, AD transformation status, and 35 characteristics, and evaluated the prognostic significance of these characteristics in 197 samples by cox method, based on the criteria of KM-Pvalue<0.01. The top 34 miRNAs with the most obvious prognosis were selected as research objects. The predicted DEmiRNAs from the top hub 10 genes were intersected with the 34 DEmiRNAs from blood samples, and a total of 3 CDEmiRNA were identified. Interestingly, the three DEmiRNAs (hsa-miR-422a, hsa-miR-4784, and hsa-miR-3944-3p) are also bound to hsa_circ_002048. The transformation curves of the three ADs are shown in Figure 10. Moreover, we found that their target genes are all *AP2M1* (Figure 11 C). The base sequence matches of the six groups are shown in Supplementary Figure S1 Figures 11 A, B. The secondary structure of AP2M1 is in Supplementary Material 1. In KEGG gene sets, GSEA results show that *AP2M1* is mainly enriched in glycosphingolipid biosynthetic ganglion series, Parkinson’s disease, Alzheimer’s disease, β-Alanine metabolism, and axonal guidance mechanism. In the database of all microRNA targets, GSEA evaluated related pathways and the results showed that hsa-miR-422a and hsa-miR-4784 and their target gene *AP2M1* may be co-enriched in the same molecular machinery (Figure 11 D, E).

A growing number of studies have demonstrated the potential impact of AP2M1 on neurodegenerative disease. *AP2M1* is a core component of the clathrin-mediated endocytosis machinery.

**FIGURE 12:**
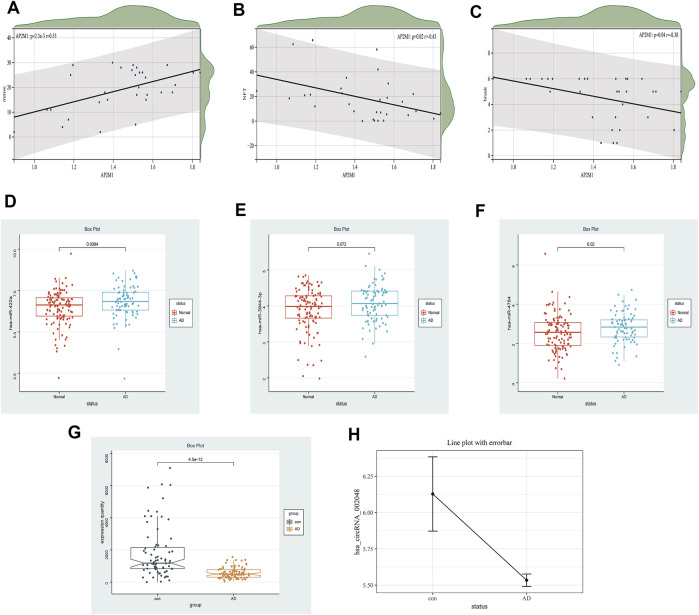
Clinical correlation and differential expression, Panel **(A–C)**. The correlation between AP2M1 and clinical information is in the GSE1297 database **(A)** AP2M1 and mmse, **(B)** AP2M1 and NFT, **(C)** AP2M1 and braak. Panel **(D–H)** Are the differential expression of miRNA, mRNA, and circRNA in a normal group compared with the AD group, respectively **(D)** hsa-miR-422a (GSE150693), **(E)** hsa-miR-3944-3p (GSE150693), **(F)** hsa-miR-4784 (GSE150693), **(G)** AP2M1 (GSE5281), **(H)** hsa_circ_002048 (only six data in GSE161435 database).

Early studies have shown that *LRK2*-dependent phosphorylation of *AP2M1* mediated dopaminergic neurodegeneration in a *Drosophila* model of PD (Liu et al., 2021). Planar cell polarity protein Vangl2 and its interacting protein *Ap2M1* regulate dendritic branching in cortical neurons (Yasumura et al., 2021). Other studies have shown that the potential candidate gene, *AP2M1*, is microdeletion causing neurodevelopmental abnormalities (Barua et al., 2022). The MiniMental State Examination (MMSE) is a reliable index of AD-related cognitive status at a given point in time (Clark et al., 1999) Postmortem scores on AD-related pathologic indices for Braak staging, hippocampal NFTs, and diffuse and neuritic senile plaques were determined as described (Geddes et al., 1997). Thus, the MMSE, Braak, and NFT values were selected as our primary markers for quantifying AD progression (Hyman, 1997; Schmitt et al., 2000; Mitchell et al., 2002). Because NFT scores increase and MMSE scores decrease with AD severity, genes up-regulated with AD could only correlate positively with NFT scores or negatively with the MMSE. Whereas genes down-regulated with AD could only correlate positively with the MMSE or negatively with NFT scores, Braak and NFT are positively correlated (Blalock et al., 2004). In our study, a significant down-regulated *AP2M1* gene was positively correlated with mmse with a correlation coefficient of 0.53 and negatively correlated with NFT with a correlation factor of −0.43, Meantime, it is also negatively correlated with Braak, the correlation coefficient is −0.38, and their *p* < 0.05 (Figure 12 A-C). Coincidentally, in 2021, researchers found that up-regulated hsa-miR-484 and hsa-miR-625-5p may inhibit the expression of *AP2M1*, thereby affecting AD (Li et al., 2021). *AP2M1* is mainly enriched in the brain and frontal cortex by RNA-Seq Expression Data (Supplementary Figure S2). In conclusion, we suspect that *AP2M1* may be regulated by the circRNA-miRNA-mRNA network, which has an important impact on Alzheimer’s disease.

Endocytosis is the process of translocation of extracellular substances into the cell through the deformation of the plasma membrane. There are three types of endocytosis: phagocytosis, pinocytosis, and receptor-mediated endocytosis depending on the size of the substance entering the cell and the mechanism of entry. Clathrin is one of the main proteins involved in endocytosis, and *AP2M1* is involved in Clathrin-dependent endocytosis (including autophagy). In earlier studies, it was found that in LC3-mediated autophagy (LANDO), the lack of LANDO in the Bone marrow chamber or specifically in microglia, typical autophagy is altered and can trigger inflammation, which is associated with β-amyloid deposition and tau hyperphosphorylation (Heckmann et al., 2019). Aberrantly phosphorylated tau protein is a major component of neurogenic fibrillary tangles in the brain in Alzheimer’s disease (AD). However, increased expression of the glycogen synthase kinase *GSK3B* is closely associated with neurogenic fibrillary tangles (Zhang et al., 2011). On my STRING website, we can observe that *MAPT*, *GSK3B*, and subunits of the *AP-2* complex, including *AP2M1*, are closely related (Supplementary Material 3). Among them, *AP2M1* and *MAPT* are co-expressed and some known experimentally determined interactions (Lee et al., 2013; Freeze et al., 2018). And they are mainly enriched in the GO terms, including Cell-cell signaling, Cellular protein metabolic process, Transport, Cellular component organization, and Wnt signaling pathway (Supplementary Material 4). In our study, we speculate that *AP2M1* expression may be inhibited due to the regulation of the circRNA-miRNA-mRNA network, thus affecting endocytosis. This may lead to a lack of normal autophagic activity associated with *AP2M1* in the bone marrow compartment or especially in microglia, rather than typical autophagy, and would result in a significant increase in pro-inflammatory cytokines, hippocampal production, and increased levels of neurotoxic β-amyloid. This inflammation is associated with β-amyloid deposition and tau hyperphosphorylation, which induces AD disease.

The limitations of the current research are still apparent. Firstly, the sample size of GSE161435 used for the analysis was relatively small with only 6 sample sets. Thus, more clinical samples are required to confirm hub circRNAs in the future. Moreover, it is necessary to validate not only the relationship between mRNA and clinical information but also the relationship between key circRNAs and miRNAs like NFTs and MMSE. Furthermore, in the ENCORI database, we used a circRNA-miRNA-mRNA competition network, but the possible incomplete coverage of various interactions could give some biased predictions. Fortunately, we found that down-regulated hsa_circ_002048 resulted in up-regulation of hsa-miR-422a, hsa-miR-4784, and hsa-miR-3944-3p, thereby inhibiting the expression of *AP2M1*, the changes in their expression levels are shown in Figure 12 D-H. On the one hand, using machine learning models including machine learning-based models and hypergeometric distribution models may better screen circRNA-miRNA-mRNA regulatory networks (Lu et al., 2020; Wang and Lei, 2020; Wang et al., 2021). On the other hand, using compression network analysis on the same data platform may further verify the competitive relationship between encircRNA-miRNA-mRNA.These will also become our future tasks. We need further studies to confirm our findings.

## Conclusion

Aberrant mRNAs and abnormal ncRNAs were identified in blood by analyzing published AD data sets. Using base sequence matching and ceRNA theory, we identified 4 blood-related ncRNAs and constructed the corresponding circRNA-miRNA-mRNA network. Down-regulating hsa_circ_002048 leads to the up-regulation of three miRNAs (has-miR-422a, hsa-miR-4784, and hsa-miR-3944-3p), which may limit the expression of *AP2M1*. The loss of *AP2M1* impairs endocytosis, including autophagy, leading to an increase in proinflammatory cytokines and neurotoxic β-amyloid levels. The resulting inflammation in turn causes β-amyloid deposition and tau hyperphosphorylation, thereby influencing AD disease. Our study may contribute to a deeper understanding of the pathogenesis of AD. These ncRNAs have the potential to serve as potential targets for diagnostic biomarkers, resulting in breakthroughs in the diagnosis and treatment of AD. However, this research is only processed bioinformatics mining and is supported by existing experiments. Our analytical results require more sample support and clinical laboratory validation.

## Data Availability

The original contributions presented in the study are included in the article/Supplementary Material, further inquiries can be directed to the corresponding author.
